# 

**Published:** 1904-02

**Authors:** 


### BOOKS RECEIVED.

Lea's Medical Epitome Series. Organic and Physiologic Chemistry. A Manual for Students and Practitioners. By A. McGlannan, M. D., Associate Professor of Physiologic Chemistry, Instructor in Clinical Laboratory, College of Physicians and Surgeons, Baltimore, Md. Duodecimo, pp. 246. Illustrated with 9 engravings. Series edited by V. C. Pedersen, A. M., M. D., Instructor in Surgery at the New York Polyclinic Medical School and Hospital. Lea Brothers & Co., Philadelphia and New York. 1903. (Price, $1.00.)

The Blues, (Splanchnic Neurasthenia), Causes and Cure. By Albert Abrams, A. M., M. D., (Heidelberg), F. R. M. S., Consulting Physician, Denver National Hospital for Consumptives, the Mount Zion and the French Hospitals, San Francisco. Duodecimo, pp. 240. Illustrated. New York: E. B. Treat & Co. 1904. (Price, $1.50.)

Transactions of the American Gynecological Society. Volume 28. For the year 1903. Twenty-eighth annual meeting held at Washington, D. C., May 12-14, 1903. J. Riddle Goffe, M. D., Secretary. Philadelphia: Wm. J. Dornan, Printer.

The Treatment of Fractures: With Notes upon a Few Common Dislocations. By Chas. L. Scudder, M. D., Surgeon to the Massachusetts General Hospital. Fourth Edition. Thoroughly Revised, Enlarged, and Reset. Octavo volume of 534 pages, with nearly 700 original illustrations. Philadelphia, New York, London: W. B. Saunders & Company. 1903. (Polished Buckram, $5.00 net; Sheep or Half Morocco, $6.00 net.)

A Textbook of Legal Medicine and Toxicology. Edited by Frederick Peterson, M. D., Chief of Clinic, Nervous Department of the College of Physicians and Surgeons, New York; and Walter S. Haines, M. D., Professor of Chemistry, Pharmacy, and Toxicology, Rush Medical College, in affiliation with the University of Chicago. Two imperial octavo volumes of about 750 pages each, fully illustrated. Philadelphia, New York, London: W. B. Saunders & Company. 1903. (Per volume, Cloth, $5.00 net; Sheep or Half Morocco, $6.00 net.)

Transactions of the National Association of United States Pension Examining Surgeons. Second annual meeting held at Washington, D. C., May 13 and 14, 1903, including an account of first meeting held at Saratoga Springs, June 9, 1902. Wheelock Rider, M. D., Secretary. Published by the Association.

The Pathogenic Microbes. By M. Le Dr. P. Jousset, Physician to the Hospital St. Jacques, Paris. Authorised translation by Horace P. Holmes, M. D. One hundred and ninety-two pages. Philadelphia: Boericke & Tafel. 1903. (Cloth, $1.00.)

The Practical Care of the Baby. By Theron Wendell Kilmer, M. D., Associate Professor of Diseases of Children in the New York School of Clinical Medicines; Assistant Physician to the Out-Patient Department of the Babies' Hospital, New York. Duodecimo, pp. 170, with 68 illustrations. Philadelphia: F. A. Davis Company. 1903. ($1.00, net, delivered.)

The Self-Cure of Consumption Without Medicine. With a Chapter on the Prevention of Consumption and Other Diseases. By Chas. H. Stanley Davis, M. D., Meriden, Conn. Pages, 176. New York: E. B. Treat & Co. 1904. (Price, 75 cents.)

The Practical Medicine Series of Year Books. Ten volumes. Issued under the general editorial charge of Gustavus P. Head, M. D., Professor of Laryngology and Rhinology in the Chicago Post-Graduate Medical School. Volume III. The Eye, Ear, Nose, and Throat. Edited by Casey A. Wood, M. D., Albert H. Andrews, M. D., and Gustavus P. Head, M. D. Duodecimo, 332 pages. Chicago: The Year Book Publishers. 1903. (Price, $1.50; entire series, $5.50, payable in advance.)

Social Diseases and Marriage. Social Prophylaxis. By Prince A. Morrow, M. D., Emeritus Professor of Genitourinary Diseases in the University and Bellevue Hospital Medical College, New York; Surgeon to the City Hospital. Octavo, pp. 390. Lea Brothers & Co., New York and Philadelphia. 1904.

